# A Case of Navigating Autism and Atypical Rashes in Dermatology Practice

**DOI:** 10.1155/crdm/2338787

**Published:** 2025-10-18

**Authors:** Benjamin S. Kahn, Kathleen Ward, Nedyalko Ivanov, Marcus B. Goodman

**Affiliations:** ^1^Goodman Dermatology, Philadelphia College of Osteopathic Medicine, Roswell, Georgia, USA; ^2^Philadelphia College of Osteopathic Medicine, Philadelphia, Pennsylvania, USA

**Keywords:** autism spectrum disorder, autistic spectrum disorder, irritant dermatitis, pediatric dermatology, pseudoverrucous papules and nodules

## Abstract

A 4-year-old nonverbal autistic female presented to the clinic with a 3-month history of a persistent, inflamed papular rash, which was violaceous to skin-toned, verrucous, and dome-shaped with crateriform ulcerations on an erythematous base localized in the perianal region. A dermatologic condition with these characteristics, location, and demographic warrants a thorough workup, physical exam, and broad differential diagnosis. The clinical appearance of these lesions can mimic many cutaneous conditions in this age group and requires careful attention for potential signs of abuse. As demonstrated in our case, with a comprehensive patient history, a biopsy, and a culture of the lesions, one can properly direct management of what looked like a broad complex differential to something more benign and underappreciated. When seeing and performing skin check screenings on particular vulnerable patient populations, such as those with autism, educating these patients and their parents is a very important aspect of management. Narrowing the condition down, we reached a diagnosis of pseudoverrucous papules and nodules, a skin rash rarely discovered in children but detected in elderly patients who are debilitated and bedridden with urinary and/or fecal incontinence. This complex case illustrates the importance of proper patient care in patients with autism and considering the entire clinical context before making a diagnosis or conclusion, specifically in vulnerable youth.

## 1. Introduction

Children with autism spectrum disorder (ASD) often present with unique dermatologic challenges influenced by their sensory differences, behavioral patterns, and communication limitations. Hyposensitivity, common in ASD, will often delay the recognition of discomfort or irritation, while urinary and fecal incontinence further predisposes autistic children to perianal and other dermatologic conditions. Perianal rashes in pediatric patients warrant a comprehensive evaluation as their etiology can range from irritant contact and diaper dermatitis to viral, yeast, or bacterial infections. In some cases, rarer conditions such as pseudoverrucous papules and nodules (PPN) may be considered. Child abuse must remain a critical consideration when evaluating unexplained or atypical presentations in the genital and perianal regions of children.

Diaper dermatitis is a common dermatologic condition among infants. Sustained exposure to moist environments increases frictional damage, compromises the skin barrier, and fosters pathogen growth. While irritant contact dermatitis is the most common presentation, chronic urinary and fecal incontinence in children with ASD may result in more severe or atypical conditions such as pseudoverrucous PPN [1]. These flat-topped skin colored papules develop in the perianal region and are more often associated with elderly, bedridden individuals or patients with urostomy sites [1]. The rarity of PPN in pediatric patients highlights the importance of considering a broad differential diagnosis, particularly in patients with disabilities such as ASD. Although PPN is uncommon, the combination of incontinence and autism makes this diagnosis clinically significant. We present a case of perianal PPN in a 4-year-old female with autism and fecal and urinary incontinence.

## 2. Case Report

**Figure 1:**
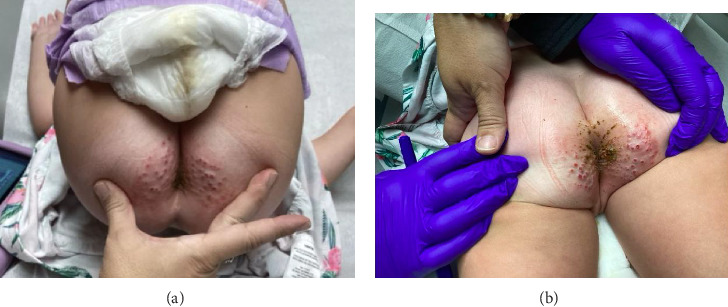
(a, b): Pseudoverrucous papules and nodules consisting of violaceous and skin-toned, dome-shaped, crateriform papules on an erythematous base. The lesions showed crust, ulceration, and secondary impetiginization located perianally and along the intergluteal cleft and buttocks.

A 4-year-old female presented with a 3-month history of violaceous and skin-toned, dome-shaped, crateriform papules on an erythematous base. The lesions showed crust, ulceration, and secondary impetiginization. They are located perianally and along the intergluteal cleft and buttocks, which are causing discomfort and pain (Figure 1). The lesions have become progressively worse over time but remain confined to this perianal region. The caregivers stated that the child's siblings and family members may have had similar lesions in the past having been exposed to her siblings with a history of molluscum contagiosum and to the shingles virus from their grandparents within the past 3 months. In the patient's previous workup, the patient was treated by her pediatrician with nystatin and Triple Paste® antifungal cream, 3 rounds of ceftriaxone injections, and cephalexin oral solution without resolution. A culture and shave biopsy of the lesion was performed.

Given the family's unsuccessful attempts to resolve the rash and the patient's unique presentation as a nonverbal autistic child, we approached the case with a fresh perspective and opted to perform a culture and biopsy of the lesion. Each of our differential diagnoses has a very different therapeutic approach, making this biopsy and culture potentially very valuable. Our differential diagnosis included molluscum contagiosum, herpes virus infections, perianal streptococcal infection, and allergic or irritant contact dermatitis. However, the possibility of a much broader differential must certainly be considered.

Starting with molluscum contagiosum, a common rash in children, can occur anywhere on the body, including the perianal region, typically spreading through skin-to-skin contact or contact with contaminated objects. These lesions are typically asymptomatic, although they can present with pruritus, erythematous, eczematous dermatitis, inflammation, pustular, or a Gianotti–Crosti-like reaction. Histopathologic examination may reveal intracytoplasmic molluscum inclusion bodies. It often resolves spontaneously within months to years, aligning with several characteristics observed in this patient [2].

We also considered a shingles or herpes virus infection, recognizing the need to rule out the possibility of a sexually transmitted infection, even in pediatric cases, and considering the patient's close contact with adult family members with shingles. Both HSV-1 and HSV-2 can present as vesicular lesions in the orofacial, genital, or perianal regions. These perigenital lesions typically present with pain, pruritus, vaginal and urethral discharge, and tender inguinal lymphadenopathy. Histopathological examination may reveal multinucleated keratinocytes and eosinophilic intranuclear inclusion bodies [2].

Additionally, streptococcal infections, common in children, may manifest with skin involvement, including the perianal area. Perianal streptococcal infections present with intense pain, erythema, dyschezia, and bloody stool. Although not tested in our patient, condyloma lata should also be considered. These lesions begin with a painless, dusky, red chancre that evolves into a papule. Secondary syphilis presents with condyloma lata, which are moist, flat papules that may have an eroded surface [2]. An RPR or treponemal test should be performed to rule out syphilis.

**Figure 2:**
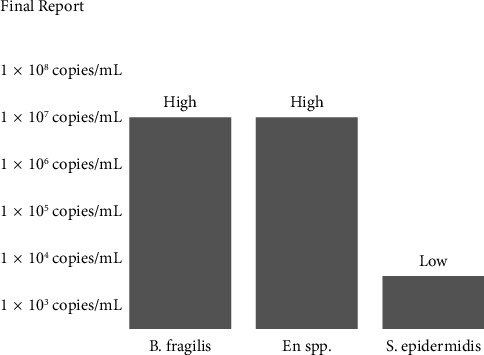
Pseudoverrucous papules and nodules culture report detected high levels of *B. fragilis*, high levels of *Enterococcus* spp., and low levels of *S. epidermidis*.

Finally, we included allergic and irritant contact dermatitis as potential causes, as the biopsy and culture were anticipated to provide definitive insights. The cultures detected high levels of *Enterococcus* spp. and *Bacteroides fragilis*, and low levels of *Staphylococcus epidermidis*, all of which are normal findings in the perianal region (Figure 2). These findings helped exclude bacterial infectious causes.

Ultimately, the biopsy findings were consistent with PPN, a type of irritant contact dermatitis, as the underlying diagnosis, guiding our treatment plan. Pathology revealed hyperkeratosis, papillomatosis, and acanthosis; features are often consistent with verruca, although typical vascular changes or inclusions were absent.

Taking the entirety of the histology along with the correct clinical context, our patient represented an underappreciated pediatric dermatitis presentation, namely, PPN. There was no evidence of Group A *Streptococcus*, herpes simplex virus, molluscum contagiosum, monkeypox, or other viral, bacterial, or fungal causes, as they had effectively been ruled out. To treat the patient's condition, we removed the irritant stimulus by educating the patient's caregivers on proper hygiene and emollients. Treatment may seem straightforward but poses a challenge in nonverbal children with ASD who struggle to express discomfort, and frustrated parents who are very concerned, yet uninformed in the scope of causes and perianal complications in this population, and struggle to be champions and vessels of communication for their autistic children.

Unfortunately, the needs of this patient population are often overlooked in medical practices and can be compounded, especially when parents are also not fully informed and educated on proper management of skincare in autistic children. We have to become better as providers, caregivers, and educators within the medical community and the handling of autistic patients and their families, and to do so, caregiver education should emphasize the importance of understanding the patient's different sensitivity reactions. Often, autistic children have sensory reactions to creams, bandages, and wet wraps, so it is important to take this into consideration and use an individualized approach while still providing adequate treatment. Furthermore, other common practices include using positive reinforcement to help engage the child in the treatment process, fostering better cooperation, and adherence may be useful [3]. In our case, the patient did not need to follow-up after the adjustments were made, as the rash was resolved in lieu of proper identification, management, and education.

## 3. Discussion

This case emphasizes the importance of considering the entire clinical context before making a diagnosis or conclusion, especially in a complex presentation, while also highlighting the vulnerability of all children, particularly those with ASD. This patient's nonverbal autism adds behavioral complexities that require careful diagnostic consideration. The presence of perianal wart-like lesions necessitates a careful evaluation including considering potential sexual child abuse. Ultimately, these factors prompted the decision to biopsy and culture the lesion to ensure an accurate diagnosis and appropriate care.

PPNs are 2- to 8-mm shiny, smooth, red, moist, flat-topped, round lesions located in the perianal region. While rare, these lesions are commonly associated with areas of chronic moisture and are observed in children with encopresis or urinary incontinence. In our case, the patient's ASD-sensory processing issues, behavioral challenges, and incontinence make this differential diagnosis particularly relevant. Often, this may be overlooked in traditional practice.

**Figure 3:**
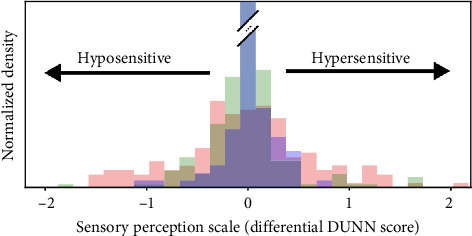
Sensory short profile scores demonstrating the distribution of mean total original SPP score per item for autistic participants (red), first-degree relatives (green), and control participants (blue). Negative scores represent hyposensory, and positive scores represent hypersensory profiles.

A hallmark of ASD is sensory processing issues. This can present as either hypersensitivity (overstimulation) or hyposensitivity (underreaction) to stimuli that typically do not bother others [4]. In our case, the patient did not react to incontinence in the way that a nonautistic individual would. In 2022, a large-scale study investigated sensory processing differences in people with ASD and examined potential genetic underpinnings. As demonstrated in Figure 3, the study found that individuals with ASD exhibit significantly greater variability in sensory processing compared to typically developing controls, with responses being both heightened and diminished. The research also identified genetic mutations in the GABAergic pathways associated with hyposensitivity, suggesting that genetic factors may contribute to sensory processing issues [4]. This is crucial when considering patient care, as individuals with ASD may be less likely to react to irritants, leading to prolonged exposure and chronic irritant dermatitis. Notably, individuals with developmental delays, including ASD, are more prone to irritant dermatitis, as they may not respond to triggers in the same way.

Our patient presented a unique case of nonverbal ASD, which posed significant challenges in communicating her desires and needs. The inability of the patient to effectively express herself to her caregivers and doctors highlights the need for healthcare providers to recognize these barriers when developing the diagnosis and management. As physicians, it is crucial to understand that ASD can present with atypical manifestations and requires an approach tailored to the patient's communication abilities. Other resources or referrals may be beneficial, such as speech therapy, occupational therapy, or referral to a specialist. Understanding the patient's underlying disabilities may be the key to unlocking the diagnosis in an otherwise complex case.

Additionally, individuals with ASD have a high prevalence of urinary incontinence. A 2019 prospective study of 47 individuals—27 adults and 20 children/teens—compared patients with ASD to a control group to investigate the prevalence of urinary incontinence. The study found that 85.1% of adults and 90% of children and teens with ASD experienced some form of incontinence [5]. Given this context, a perianal rash in our patient, such as PPN, aligns with the findings commonly observed in autistic individuals with incontinence. This further supports the hypothesis that the rash may be related to underlying urinary issues, which are notably more frequent in patients with ASD, and helps make the diagnosis of PPN more clinically cohesive.

In evaluating perianal wart-like lesions, it is important to consider child abuse as part of a thorough diagnosis. In the United States, physicians are mandated reporters and are required to report suspected child sexual abuse, including the presence of a sexually transmitted disease. In our case, child abuse was considered. However, given that such a claim warrants careful consideration, we decided it was necessary and important to take a biopsy and culture to avoid bias. Opting to take these measures, especially when clinical findings are ambiguous, has proven in this case to be extremely valuable. If a biopsy and culture were taken initially due to the complexity of the case, it would have saved our patient many hardships of trial medications and doctor's appointments.

PPN in the pediatric population is commonly encountered in children with excretory incontinence and infrequent diaper changes. A similar case was presented in a 4-year-old female with developmental delay, mild hydrocephalus, spina bifida occulta, and excretory incontinence. This patient presented with PPN in the mons, vulva, inner buttocks, and perineal area [6]. A case of a 10-month-old male with penoscrotal hypospadias had histological overlap of PPN on the scrotum as well [7]. Similarly, these lesions resolved with frequent diaper changes and petrolatum/zinc oxide ointment [6, 7]. Another case of a 4-year-old with cloacal atresia and excretory incontinence presented with a vesicular variant of PPN and secondary infection [8]. These case findings highlight the association between PPN and excretory incontinence.

Overall, this case provided valuable insight into the importance of considering all aspects of a patient case, especially in often overlooked and vulnerable populations affected with ASD in order to reach a diagnosis while avoiding bias and unnecessary hardships. Through careful and comprehensive evaluation, we were able to clarify a complex case and highlight a diagnosis that is often overlooked. This case will likely serve as a prototypical example for similar clinical contexts, particularly in cases where not all aspects of the clinical encounter are considered, thus highlighting a diagnosis that is often overlooked in practice.

## Data Availability

The dataset supporting this case report is available from the corresponding author on reasonable request.
